# EHMTI-0243. Pressure pain sensitivity maps of the head are similar in patients with unilateral or bilateral migraine

**DOI:** 10.1186/1129-2377-15-S1-E12

**Published:** 2014-09-18

**Authors:** M Ruiz, J Barón, MI Pedraza, C Rodriguez, A Guerrero, P Madeleine, ML Cuadrado, C Fernandez-de-las-Peñas

**Affiliations:** 1 Neurologist, Hospital Clínico Universitario de Valladolid, Valladolid, Spain; 2 Centre for Sensory-Motor Interaction (SMI) Department of Health Science and Technology, Aalborg University, Aalborg, Denmark; 3 Neurology, Hospital Clínico Universitario San Carlos, Madrid, Spain; 4 Departamento de Fisioterapia Terapia Ocupacional Rehabilitación y Medicina Física, Universidad Rey Juan Carlos, Alcorcon Madrid, Spain

## Introduction

Migraine is considered a unilateral headache though several patients exhibit bilateral pain. Previous studies suggest the presence of bilateral pressure pain sensitivity in temporalis region in unilateral migraine patients. No data exists concerning pressure sensitivity maps of the head in this population.

## Aim

To determine differences in pressure pain sensitivity maps of the head in patients with unilateral or bilateral migraine.

## Methods

Ten patients with strictly unilateral migraine on left side, 10 on the right and 10 with bilateral migraine, all of them episodic. Pressure pain thresholds were measured in 21 points distributed over the scalp. Locations of those points were based on normalized positions for electroencephalogram recordings. Pressure pain sensitivity topographical maps were constructed. All patients were headache-free when evaluated.

## Results

No differences existed among the 3 groups in age, time from onset of migraine and days with pain during previous month. Cartographic maps of the head did not reveal significant differences between patients with predominantly unilateral migraine (either left or right side dominant) and bilateral migraine (all, P>0.494). Pressure sensitivity maps of the head revealed that the temporal part of the head (points F8, F7, F4, F3) were more sensitive (all, P<0.01) than the remaining parts of the head.

## Conclusion

Our results suggest that pressure pain sensitivity map of the head is independent of the presence of bilateral or unilateral pain in migraine. Further, it seems that the temporal scalp may be the most sensitized part of the head in these patients, accordingly to previous studies.

No conflict of interest.

